# Editorial: Complementary and Alternative Medicine Use in Cancer Patients

**DOI:** 10.3389/fnut.2022.875937

**Published:** 2022-03-23

**Authors:** Kathryn T. Knecht, Amy L. Stockert

**Affiliations:** ^1^Department of Pharmaceutical and Administrative Sciences, Loma Linda University School of Pharmacy, Loma Linda University, Loma Linda, CA, United States; ^2^Department of Pharmaceutical and Biomedical Sciences, Raabe College of Pharmacy, Ohio Northern University, Ada, OH, United States

**Keywords:** cancer, nutrition, ketogenic, probiotic, glioblastoma, wound healing, dietary supplements

This Research Topic highlights Complementary and Alternative Medicine (CAM) nutritional approaches in the treatment of cancer. Nutritional approaches are an important aspect of CAM and include dietary regimens, nutrient intake, consumption of phytochemicals, and use of dietary supplements. In the treatment of patients, mechanisms that might directly alter cancer growth and progression are of keen interest (1). However, indirect effects should also be considered. For example, treatments such as surgery and chemotherapy have their own adverse effects on the patient, and dietary approaches could play an indirect role in mitigating these effects and/or enhancing patient recovery and wellbeing. Additionally, cancer could also contribute to patient malnutrition and therefore incur additional nutritional requirements that could be alleviated by CAM approaches. This Editorial will highlight examples of direct and indirect nutritional approaches in cancer treatment, especially highlighting effects of ketogenic diet on glioblastoma and benefits of nutritional support during anorexia/cachexia, wound healing and recovery from cancer surgery.

The use of ketogenic diets is of interest in cancer treatment (2, 3), particularly in glioblastoma for which treatment options are limited (4). The metabolic needs of cancer cells are thought to be different from those of normal cells, and as such could be targeted while minimizing effects on normal tissue. Cancer cells require high levels of energy to support rapid metabolism and cell division. The Warburg effect describes the preference of cancer cells for glycolysis vs. oxidative phosphorylation in utilizing glucose, even if oxygen is present (aerobic glycolysis). Because glycolysis is a glucose-inefficient process, these cancer cells would have a high glucose requirement, and elevated glucose levels in the body thus could facilitate cancer cell growth. Ketogenic diets, which decrease glucose consumption while increasing lipid consumption, produce ketone bodies that can be used by normal cells but not by cancer cells and consequently decrease energy availability in cancer cells. Montella et al. review oncogenic adaptations in glioblastoma metabolism that trend toward aerobic metabolism; the variable metabolic processes occurring in glioma cells; the therapeutic use and limitations of a ketogenic diet; and a possible role of gut microflora in ketogenic cancer treatment.

Seyfried et al. describe a case of a glioblastoma patient who rejected standard treatment of radiation and chemotherapy and instead adopted Ketogenic Metabolic Therapy followed by surgical debulking. The patient was both knowledgeable and motivated in following and monitoring a strict reduced calorie ketogenic diet, accompanied by an exercise regimen, breathing exercises, stress reduction, and dietary supplements including vitamins, minerals, turmeric, resveratrol, omega 3 fatty acids, and boswellia. The patient experienced unusually slow tumor growth with one period of faster progression that closely followed a relaxation in diet adherence. At the time of writing, the patient had pursued this therapy for 80 months.

Just as poor nutrition might increase the vulnerability of patients to cancer risk, so might malnutrition be a consequence of cancer-induced anorexia and cachexia. Malnutrition can in turn impair patients' ability to respond to both the disease and to the added stress of cancer treatment (5). Duan et al. illustrate the value of overall nutritional support by showing that oral nutritional supplementation added to megasterol acetate for anorexia/cachexia in lung cancer patients increased Body Mass Index (BMI), Eastern Cooperative Oncology Group (ECOG) score (indicating patient functionality), and serum albumin and pre-albumin levels. In contrast, beta-hydroxy methyl-butyl butyrate/arginine/glutamine or fish oil had not been reported to improve body weight compared to megasterol alone.

Nutritional support can be especially important with surgery, which is a mainstay of treatment of solid cancers. Decreased wound healing and susceptibility to infection could impair recovery in surgery, and these processes could be altered by dietary components. The effects of hyperglycemia in facilitating the growth of cancer cells has been described, but conversely hyperglycemia can have adverse effects on normal cells and can impede the process of healing. In this regard, Lan et al. demonstrate that hyperglycemia in non-diabetic patients can impair recovery from gastrectomy for gastric cancer, noting increased infection and sepsis. It would be of interest to further examine peri-operative use of a ketogenic diet in this light.

On the other hand, nutritional CAM could promote recovery. Pancreaticoduodenectomy, a complex surgery used to treat pancreatic cancers, has a high (60%) risk of infection and other complications that can lead to longer hospital stays and increased mortality. Beneficial probiotic microorganisms can decrease the growth and translocation of harmful bacteria and strengthen immunity. Prebiotics are substances that increase probiotic growth, and synbiotics are formulated to contain both probiotics and prebiotics (6). In a meta-analysis, Tang et al. show that the use of probiotics or synbiotics decreased postoperative infection and delay in gastric emptying, as well as shortening hospital stay and antibiotic duration. However, mortality rate was not affected. Due to the fairly small number of studies, the authors point out the need for further studies of efficacy as well as safety.

Interestingly, non-healing chronic wounds can promote cancer formation (7), and thus increased wound healing might not only be a benefit to post-surgical patients but also play a benefit in the prevention of cancer. Monika et al. elaborate upon the multiple biological processes of wound healing and describe a possible role for natural products and phytochemicals, including *Lawsonia inermis, Azadirachta indica*, and neem in facilitating the healing process.

Gu et al. highlight some of the challenges in examining effects of nutritional CAM. This group surveyed women diagnosed with ovarian cancer in regard to their use of dietary supplements in the year prior to diagnosis. They found no relationship between pre-diagnosis overall supplement use and ovarian cancer survival, nor any relationship with the specific supplements vitamin A, vitamin C, vitamin D, vitamin E, multivitamins, calcium, and fish oil/DHA. In fact, use of vitamin B was associated with decreased survival. Their observations are in line with mixed results in the existing literature on this Research Topic, despite multiple theoretical benefits of these nutrients (8). However, they point out that some supplement users might have used supplements to address preexisting health conditions or nutritional deficiency, making interpretation more difficult.

Possible applications of CAM in cancer are many and varied, affecting prevention, treatment and adjunct treatment, and patient recovery and wellbeing. This Research Topic suggests several areas of approach as well as highlighting the need for more research on these subjects.

## Author Contributions

KK wrote and edited the initial draft of the manuscript. AS compiled reference material and reviewed and edited the final version. Both authors contributed to the article and approved the submitted version.

## Conflict of Interest

The authors declare that the research was conducted in the absence of any commercial or financial relationships that could be construed as a potential conflict of interest.

## Publisher's Note

All claims expressed in this article are solely those of the authors and do not necessarily represent those of their affiliated organizations, or those of the publisher, the editors and the reviewers. Any product that may be evaluated in this article, or claim that may be made by its manufacturer, is not guaranteed or endorsed by the publisher.

## References

[1] Jia L. Cancer complementary and alternative medicine research at the US National Cancer Institute. Chin J Integr Med. (2012) 18:325–32. 10.1007/s11655-011-0950-5 22241505

[2] Murakami M Tognini P. Molecular mechanisms underlying the bioactive properties of a ketogenic diet. Nutrients. (2022) 14:782. 10.3390/nu14040782 35215432 PMC8879219

[3] Tulipan J Kofler B. Implementation of a low-carbohydrate diet improves the quality of life of cancer patients - An online survey. Front Nutr. (2021) 8:661253. 10.3389/fnut.2021.661253 34458297 PMC8384958

[4] Bryukhovetskiy I. Cell-based immunotherapy of glioblastoma multiforme. Oncol Lett. (2022) 23:133. 10.3892/ol.2022.13253 35251352 PMC8895466

[5] Molfino A Imbimbo G Laviano A. Current screening methods for the risk or presence of malnutrition in cancer patients. Cancer Manag Res. (2022) 14:561–7. 10.2147/CMAR.S294105 35210853 PMC8857947

[6] Gebrayel P Nicco C Al Khodor S Bilinski J Caselli E Comelli EM. Microbiota medicine: towards clinical revolution. J Transl Med. (2022) 20:111. 10.1186/s12967-022-03296-9 35255932 PMC8900094

[7] Rybinski B Franco-Barraza J Cukierman E. The wound healing, chronic fibrosis, and cancer progression triad. Physiol Genomics. (2014) 46:223–44. 10.1152/physiolgenomics.00158.2013 24520152 PMC4035661

[8] Ciebiera M Esfandyari S Siblini H Prince L Elkados H Wojtyla C. Nutrition in gynecological diseases: Current perspectives. Nutrients. (2021) 13:1178. 10.3390/nu13041178 33918317 PMC8065992

